# Loss of Novel Diversity in Human Gut Microbiota Associated with Ongoing Urbanization in China

**DOI:** 10.1128/msystems.00200-22

**Published:** 2022-06-21

**Authors:** Shan Sun, Huijun Wang, Annie Green Howard, Jiguo Zhang, Chang Su, Zhihong Wang, Shufa Du, Anthony A. Fodor, Penny Gordon-Larsen, Bing Zhang

**Affiliations:** a Department of Bioinformatics and Genomics, College of Computing and Informatics, UNC Charlotte, Charlotte, North Carolina, USA; b National Institute for Nutrition and Health, Chinese Center for Disease Control and Prevention, Beijing, China; c Carolina Population Center, UNC Chapel Hill, Chapel Hill, North Carolina, USA; d Department of Biostatistics, Gillings School of Global Public Health, UNC Chapel Hill, Chapel Hill, North Carolina, USA; e Department of Nutrition, Gillings School of Global Public Health, UNC Chapel Hill, Chapel Hill, North Carolina, USA; Pacific Northwest National Laboratory

**Keywords:** gut microbiota, metagenomics, urbanization, diversity

## Abstract

Recent rapid and large-scale urbanization has had a profound impact on human lifestyles and is associated with an increased risk of many diseases. Recent studies have revealed large differences in the human gut microbiota across populations in countries at different stages of urbanization. However, few studies have analyzed the impact of ongoing urbanization within the same geographic region. In this study, we sampled 214 participants in communities of different urbanization levels within two provinces of China and reconstructed draft prokaryotic genomes with metagenome sequences. The genomes were clustered into 447 species-level operational taxonomic units (OTUs), among which 196 did not have genomes in public reference databases according to the GTDB-Tk pipeline. The novel OTUs comprised 19.1% abundance in rural participants and 16.0% in urban participants, increasing the proportion of classified reads from 47.6% to 65.3% across all samples. Among the unknown OTUs, 26 OTUs present in rural samples were absent in urban participants, while 70 unknown OTUs were more abundant in rural than urban participants, suggesting potential loss and growth suppression of novel human symbionts during urbanization. Moreover, there were a higher number of genes, especially transporters, identified in genomes assembled from urban samples. This change in gene functionality indicates that urbanization not only altered the community structure of the human gut microbiota but also impacted its functional capacity. Taken together, these data show a dramatic change in the microbiota with urbanization and suggest the importance of cataloging microbial diversity from rural populations while these communities still exist.

**IMPORTANCE** Previous studies have reported the differences in human gut microbiota across populations of different urbanization levels, but most of the studies focused on populations across different geographic regions. In this study, we analyzed the impact of ongoing urbanization in neighborhoods within the same geographic region. By assembling shotgun metagenome sequences, we reconstructed prokaryotic genomes from human gut microbiota and found that the novel bacterial OTUs were less abundant and less prevalent in urban participants than in rural participants, indicating potential loss and suppression of novel human symbionts during urbanization. Genes, including transporters and antibiotic resistance genes, were enriched in genomes of urban origins, suggesting change in functional potential of the microbiota. These findings suggest the significant influence of urbanization on human gut microbiota and the necessity of exploring the microbial diversity of rural populations.

## INTRODUCTION

In the last few decades, the world has experienced rapid and large-scale urbanization, and this transformation from rural to highly urbanized communities is projected to accelerate into the future. The 2018 Revision of World Urbanization Prospects from the United Nations reported that the urban population increased from 751 million to 4.2 billion (30% to 55% of the world population) between 1950 and 2018 and that urbanization combined with world population growth could increase urban population by another 2.5 billion people by 2050 (1). Urbanization is a complicated process that changes many aspects of the environment, including behaviors, lifestyles, and access to goods and services, all of which alter exposure patterns. These changes that accompany urbanization are associated with increased risks of diseases, including obesity, diabetes, hypertension, cardiovascular disease, and cancer (2–5). The human microbiota plays essential roles in host health, and its dysbiosis is associated with many diseases. Our group and others have previously reported that urbanization is associated with microbiota shifts in both taxonomy and functions such as increased *Bacteroides*/*Prevotella* ratios, overrepresentation of functional genes related to degradation of amino acids and simple sugars, loss of functions associated with plant fiber degradation, and an increasingly urbanized microbiota (6–8).

With the ongoing massive and uneven urbanization in China, people with highly traditional (rural) and highly urbanized lifestyles often reside in adjacent areas within the same region. This provides a chance to investigate the influence of ongoing urbanization while controlling for geographic regional factors and ethnic backgrounds that are often confounding factors for urbanization studies. Our previous study in Hunan Province of China compared 20 recently urbanized subjects to 20 rural subjects and found that the taxonomic composition of urban population microbiota has shifted to greater similarity to that of American subjects and an increased abundance of antibiotic resistance and virulence genes (8).

The recent development of high-throughput genomic approaches has facilitated the recovery of microbial genomes from shotgun metagenome sequencing samples (9–11). In this study, we sampled rural and recently urbanized populations in Hunan Province and Guizhou Province in China and collected metagenome samples of 214 participants. With newly established metagenomic assembly and binning approaches, we reconstructed microbial genomes of urban and rural origins and found many novel microbial species that were not in the current microbial genome databases. These novel species were more prevalent and more abundant in the gut microbiota of rural populations, and this is likely related to the sampling bias toward urban samples in current databases. The identification of novel species relative to current databases points to the importance of sampling and investigating these unexplored species in rural populations. These results represent a very detailed view of urbanization differences in the taxonomic and functional structure of assembled genomes of the microbial community. Our results suggest that urbanization is associated with loss and decline of novel human symbionts in the local population as well as significant changes in taxonomic profile and gene function.

## RESULTS

### Urban genomes are larger, better annotated, and better represented in current microbial databases than rural genomes.

**FIG 1:**
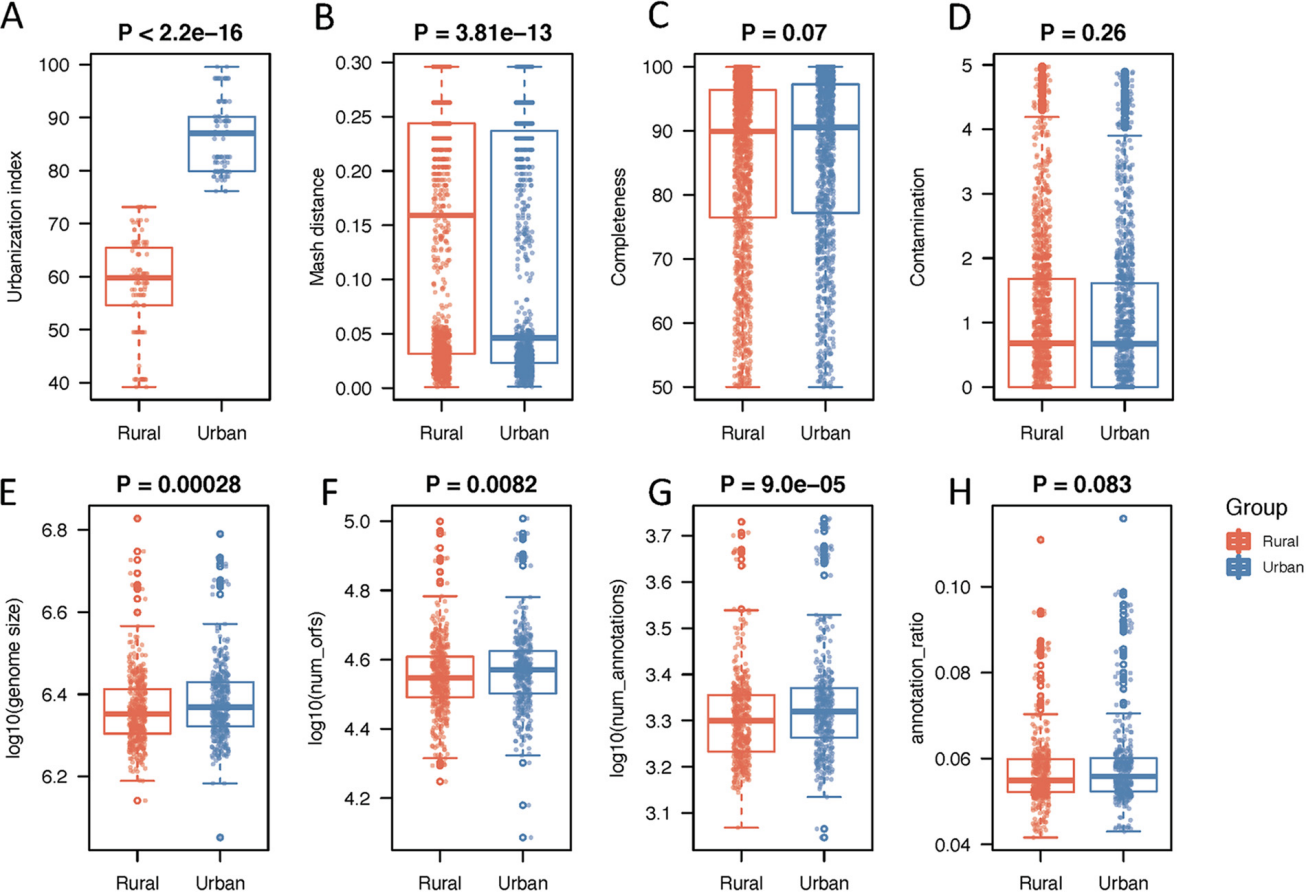
Characteristics of metagenome-assembled genomes (MAGs). (A) Urbanization index difference between urban and rural participants. (B) Mash distances to the RefSeq database of the MAGs assembled from urban and rural samples. (C and D) Completeness and contamination of MAGs assembled from urban and rural samples. (E to H) Genome size (log_10_), number of ORFs (log_10_), number of annotations (log_10_), and the annotation/ORFs ratios of the MAGs assembled from urban and rural samples. The differences were analyzed with Wilcoxon’s test.

We used an assembly approach to reconstruct genomes from metagenome samples collected as part of the China Health and Nutrition Survey, which was designed to investigate the impact of urbanization on population health. The 214 surveyed participants were from areas of different urbanization levels within two provinces (Fig. 1A). Figure 1A visualizes the relationships between urban and rural participants and the urbanization index. Variables potentially related to urbanization and the human microbiome are shown in Table 1. Education, daily physical activity, toilet facilities, daily fiber intake, and drinking water source were significantly different between rural and urban participants. The sequencing depth of all samples was 45,880,309 reads/sample on average (from 39,644,752 to 55,581,836 reads), with 46,808,343 reads/sample for urban samples and 45,050,827 reads/sample for rural samples (*t* test, *P* = 7.1E-4). While this difference is statistically significant, we do not believe that the 3.8% difference in sequencing depth is large enough to explain our results or cause an unbalanced comparison between rural and urban samples. We used a single-sample assembly strategy and reconstructed 5,594 microbial metagenome-assembled genomes (MAGs). After filtering low-quality MAGs (<50% completeness or >5% contamination), 843 MAGs were considered high quality (>90% completeness, <5% contamination, and 0% strain heterogeneity), and 1,675 MAGs (>50% completeness and <5% contamination) were considered medium quality. Among these 2,518 high- and medium-quality MAGs, 1,395 MAGs were assembled from 113 rural samples (12.3 MAGs/sample), and 1,123 MAGs were assembled from 101 urban samples (11.1 MAGs/sample) (*t* test, *P* = 0.077). The average genome size was 2,198,904 bp for MAGs retrieved from metagenomes, with 2,246,518 bp for urban MAGs and 2,160,573 bp for rural MAGs.

**TABLE 1:** Characteristics of study participants

Characteristic	Data for:		*P*
	Urban	Rural	
No. of participants	101	113	
Age (yrs)	52.2 (9.9)	51.6 (9.4)^*a*^	0.58
Female (%)	57.4	64.3^*a*^	0.33
Education (%)			1.1E-7
Less than primary school	12.9	31.3^*a*^	
Completed primary school	11.9	17.9^*a*^	
Completed secondary	42.6	46.4^*a*^	
Graduate or technical school	32.7	4.5^*a*^	
BMI	24.6 (3.3)	24.2 (6.9)^*a*^	0.40
Daily physical activity estimation^*c*^	100.3 (101.1)^*a*^	203.4 (179.9)^*a*^	5.2E-7
Daily energy intake (kcal, avg of 3 days)	2,038.6 (794.7)	1,838.3 (606.0)^*a*^	0.065
Daily fiber intake (g, avg of 3 days)	13.4 (7.8)	11.7 (8.8)^*a*^	0.018
Drinking water source (percent from source)			0.011
In-house tap	76.2	60.4^*b*^	
In-yard tap	17.8	27.0^*b*^	
In-yard well	0	6.3^*b*^	
Other	5.9	6.3^*b*^	
Toilet facilities (percent toilet type)			3.0E-4
In-house flush	94.1	57.7^*b*^	
Other	5.9	42.3^*b*^	

a Data are missing for one participant. The numbers in parentheses are standard deviations, in the format of mean (SD).

b Data are missing for two participants.

c Metabolic Equivalent of Task (MET) hours per week.

We found no differences in the quality of assembled genomes between urban and rural samples (Fig. 1C and D). We calculated the distances between our assembled genomes with genomes in the RefSeq database (release 93) with Mash (12) and found that the genomes assembled from rural samples were less represented in the RefSeq database than urban samples (Fig. 1B) and that urban and rural genomes also differed in genome size (Fig. 1E), the number of open reading frames (ORFs) (Fig. 1F), and the number of genes annotated (Fig. 1G), with the genomes assembled from urban samples having larger genomes with more annotations. We next calculated the ratio of annotated genes to predicted ORFs to correct for the influence of genome size. The ratio of annotated genes to predicted ORFs is not significantly different between urban and rural genomes (Fig. 1H).

To investigate if the observed differences in novelty with urbanization could be explained by the factors associated with urbanization and microbiome in Table 1, we next examined the relationship of these factors and the Mash distances between our assembled genomes and the genomes in the RefSeq databases. We found that education, drinking water source, toilet type, energy, and fiber intake were all associated with the similarity to the RefSeq database, while age, sex, physical activity, and BMI were not significantly associated (see Fig. S1 in the supplemental material). However, the magnitude of differences associated with these variables (with *P* values ranging from 0.27 for sex to 0.002 for toilet type) (Fig. S1) was smaller than the difference observed for urbanization (with a *P* value of 3.81E-13). We therefore chose to focus on urbanization for our downstream analyses.

10.1128/msystems.00200-22.1 FIG S1 Associations between urbanization related factors and the mash distances to database. The associations between Mash distance (see Materials and Methods) and education, drinking water source, and toilet type were tested with analysis of variance (ANOVA). The association between mash distance and sex was tested with Wilcoxon test. The associations between mash distance and age, physical activity, energy intake, fiber intake, and BMI were tested with Spearman’s correlation. Download FIG S1, PDF file, 0.6 MB. Copyright © 2022 Sun et al. 2022 Sun et al. https://creativecommons.org/licenses/by/4.0/ This content is distributed under the terms of the Creative Commons Attribution 4.0 International license.

### Genomes of unknown species revealed in metagenome-assembled genomes.

**FIG 2:**
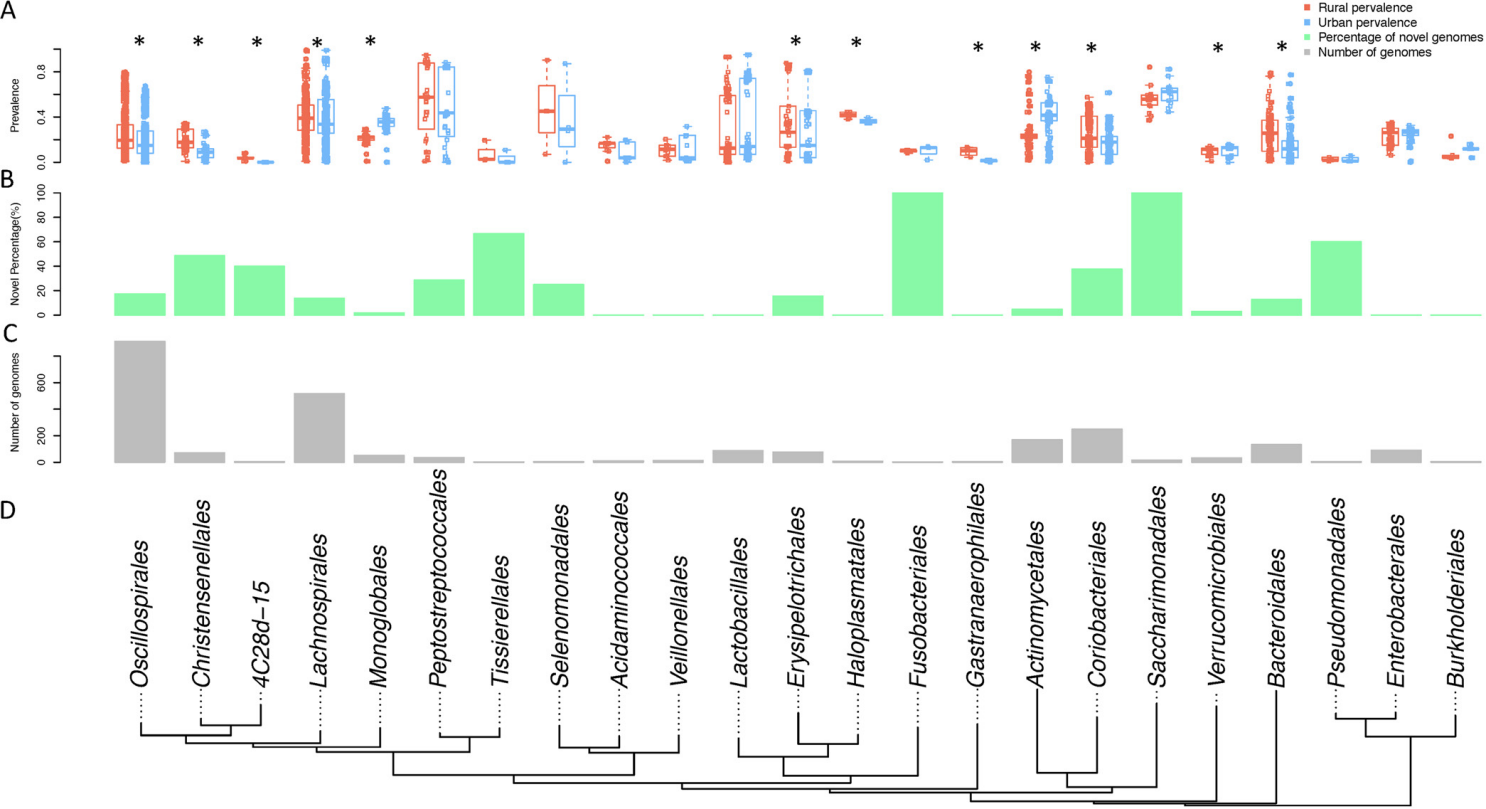
The metagenome-assembled genomes (MAGs) in this study are distributed across 23 orders (with >1 MAG). (A) The rural and urban prevalence of MAGs are different in many orders (Wilcoxon test; *, FDR < 0.1). (B) Percentage of novel MAGs in all MAGs assembled in each order. (C) Number of MAGs assembled in each order. (D) Phylogenic tree of the major orders (with >1 MAG) of MAGs assembled in this study (a subset of the Genome Taxonomy Database tree).

We used GTDB-Tk to investigate the taxonomic classification of the assembled genomes against the Genome Taxonomy Database (GTDB). Among the 2,518 genomes, 2 were considered to belong to novel families, 100 were considered as belonging to novel genera in known families, and 339 were considered novel species in known genera. With 279 novel genomes and 1,116 known genomes assembled from rural samples, 162 novel genomes, and 961 known genomes assembled from urban samples, the percentage of novel genomes is higher for rural-originated ones (20% versus 14%, Fisher’s exact test, odds ratio [OR] = 1.48, *P* = 0.00027). The assembled genomes belonged to 13 phyla, 14 classes, 33 orders, 70 families, and 180 genera. Among the 33 orders, 23 that have more than 1 member are shown in the phylogenetic tree in Fig. 2. The assembled genomes belonging to different orders showed different prevalence between urban and rural samples (Fig. 2A). Orders that include *Oscillospirales*, *Christensenellales*, and *Lachnospirales* were significantly more prevalent in rural samples, while orders that include *Actinomycetales* and *Verrucomicrobiales* were significantly more prevalent in urban samples (Fig. 2A). The order distribution of assembled genomes is shown in Fig. 2C, with *Oscillospirales* consisting of the largest number of assembled genomes, with 912 (36.2%), followed by *Lachnospirales* with 517 genomes (20.3%). The novel genomes were enriched in some orders compared to the known assembled genomes (Fig. 2B). All genomes classified to *Fusobacteriales* and “*Candidatus* Saccharimonadales” were novel, while those belonging to *Tissierellales*, *Pseudomonadales*, and *Christensenellales* were 67%, 60%, and 48% novel, respectively (Fig. 2B). We mapped all genomes back to the shotgun sequencing reads and found that genomes in *Actinomycetales*, *Monoglobales*, and *Verrucomicrobiales* were significantly more prevalent in urban samples, while 8 orders, including *Christensenellales*, *Oscillospirales*, and *Coriobacteriales*, were more prevalent in rural samples.

### Novel species are more prevalent and abundant in rural participants.

**FIG 3:**
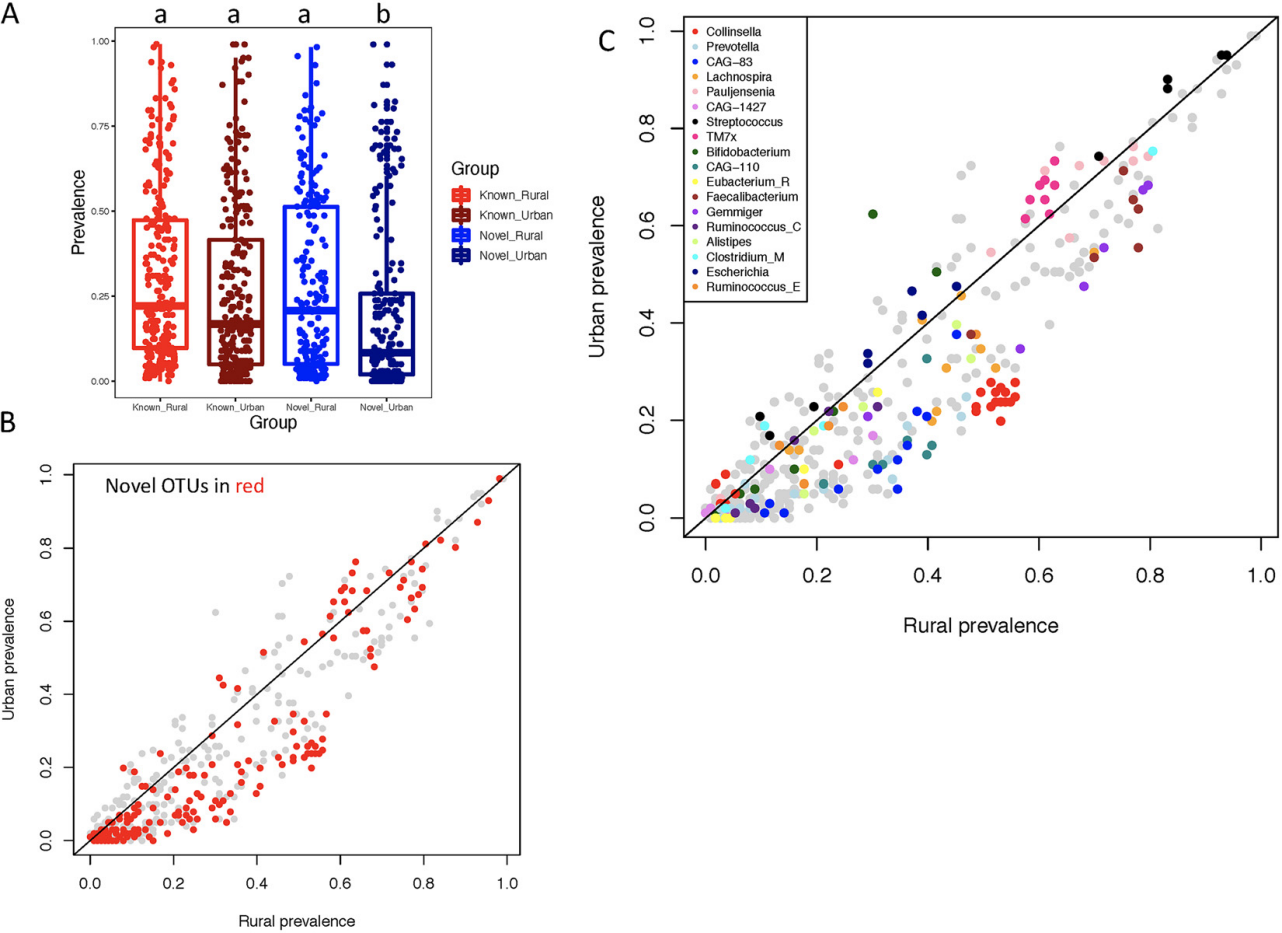
The prevalence of species-level OTUs in urban and rural participants. (A) Prevalence of the novel OTUs was significant lower in urban samples compared to rural samples (20% versus 28% on average, *P* = 5.84E-05, Wilcoxon test). (B) This trend was also observed in most novel OTUs (red) when comparing the rural and urban prevalence of each OTU, with 26 novel OTUs present in rural samples but absent in urban. (C) Concordant urbanization-related patterns in some taxonomic groups (colored by genus classification).

One possible limitation of our analysis at the level of individual MAGs is that very closely related genomes can be treated as distinct entities even if they belong to the same species. To dereplicate highly similar MAGs that could show similar patterns, we clustered the MAGs to species-level operational taxonomic units (OTUs) to create pangenomes of the same taxonomic classification. These analyses of pangenomes at the OTU level are less sensitive to very small genome changes and therefore may be more reproducible for comparison in future studies. The genomes classified to the species level were dereplicated to 251 known species (see Materials and Methods). For the novel genomes that do not have species assignment, we calculated pairwise average nucleotide identity (ANI) against those classified to the same higher taxonomic group and clustered them to species-level OTUs based on 95% ANI. With this method, the novel genomes were clustered into 196 species-level OTUs. We clustered the genomes of the same species into pangenomes and mapped the pangenomes back to the shotgun metagenome sequencing reads and calculated their relative abundance in each sample. In addition to the differential rural and urban prevalence at the MAG level (Fig. 2A), we conducted further analysis to test whether rural and urban prevalence were also different at the OTU level (Fig. 3). The relative abundance of novel OTUs was 19.1% on average across all rural samples and 16.0% across urban samples. Inclusion of the novel OTUs increased the percentage of mapped reads (calculated based on the number of reads aligned with BWA and their coverage) from 47.6% to 65.3% in the whole study. The prevalence of the novel OTUs was significantly lower in urban samples than in rural samples (20% versus 28% on average across all novel OTUs; Wilcoxon test, *P* = 5.84E-05) (Fig. 3A). This trend was also observed in most novel OTUs when comparing the rural and urban prevalence of each OTU (Fig. 3B), with 26 novel OTUs present in rural samples but absent in urban. We found concordant urbanization-related patterns in some taxonomic group. For example, OTUs in genera *Collinsella*, *Prevotella*, *CAG-83*, *Faecalibacterium*, and *Gemmiger* were generally more prevalent in rural, while those in genera TM7x, Escherichia, Streptococcus, and *Bifidobacterium* were of higher prevalence in urban participants (Fig. 3C).

**FIG 4:**
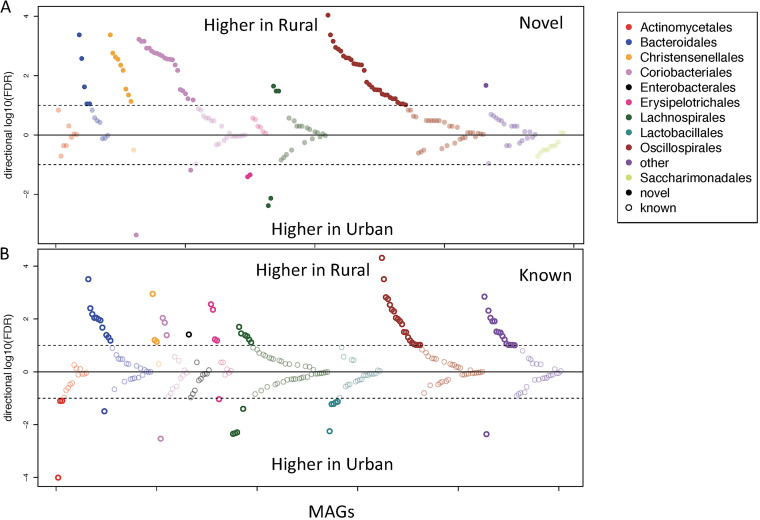
Taxonomic distribution of novel and known species-level OTUs associated with urbanization. Each point is a species-level OTU, and the *y* axis shows the log_10_ of false-discovery rates (FDRs) multiplied by the direction of association as shown in the figure. The dotted line indicates a 10% FDR. The significance was analyzed with Wilcoxon test, and the OTUs were colored based on their order classification (Wilcoxon test, FDR < 0.1).

With a univariate Wilcoxon test, 22 OTUs (6 novel and 16 known) were more abundant in urban participants, while 133 (70 novel and 63 known) were more abundant in rural participants (Fig. 4). Among the novel OTUs more abundant in rural, 16 were classified as belonging to genus *Collinsella* (p_Actinobacteriota, c_Coriobacteriia, o_Coriobacteriales, and f__Coriobacteriaceae), 7 belonged to a novel genus in family *CAG-74* (p_Firmicutes_A, c_Clostridia, and o_Christensenellales), 6 belonged to genus *CAG-83* (p_Firmicutes_A, c_Clostridia, o_Oscillospirales, and f_Oscillospiraceae), and 5 belonged to genus *CAG-110* (p_Firmicutes_A, c_Clostridia, o_Oscillospirales, and f_Oscillospiraceae), while among the OTUs more abundant in urban samples, 5 were known species in genus Streptococcus, 2 were known ones in genus *Rothia*, and another 2 were novel ones in genus *Collinsella.* Among the urban enriched species, Streptococcus pasteurianus, Streptococcus parasanguinis, Rothia mucilaginosa, and Eggerthella lenta were reported as pathogens (13–16).

In general, the order *Oscillospirales* has the most OTUs that were different between rural and urban (all enriched in rural samples, 31 novel and 20 known), followed by *Coriobacteriales* with 24 OTUs (21 novel and 3 known) more abundant in rural and 3 (2 novel and 1 known) more abundant in urban (Fig. 4). All 26 assembled OTUs in the order *Lactobacillales* were known, with 5 of them more abundant in urban and none enriched in rural. The 11 OTUs classified as *Saccharimonadales* were all novel, and their abundance was not significantly different between rural and urban samples (Fig. 4). We also analyzed the correlations between the abundance of OTUs and the urbanization index, and the trend was consistent with the test based on urban/rural status. With the Spearman’s correlation analyses, 22 OTUs (4 novel and 18 known) were positively correlated with the urbanization index (higher in urban participants), while 105 (58 novel and 47 known) were negatively correlated (Fig. S2). We compared the Shannon diversity of the urban and rural samples and found that the rural samples have significantly higher Shannon diversity than urban samples (Wilcoxon test, *P* = 0.023) (Fig. S3). We also analyzed the associations of the OTUs and the host factors in Table 1. There are OTUs significantly associated with sex (49 OTUs), drinking water source (17 OTUs), toilet type (14 OTUs), education (2 OTUs), daily fiber intake (2 OTUs), and age (1 OTU), but of a smaller number than those significantly associated with urbanization (Fig. S4 and Table S1).

10.1128/msystems.00200-22.2 FIG S2 Taxonomic distribution of novel and known species-level OTUs associated with the continuous measure of urbanization index. Each point is a species-level OTU, and the *y* axis shows Spearman’s correlation coefficients. The OTUs were colored based on their order classification, and insignificant OTUs are shown in light colors (Spearman’s correlation, FDR < 0.1). Download FIG S2, PDF file, 0.1 MB. Copyright © 2022 Sun et al. 2022 Sun et al. https://creativecommons.org/licenses/by/4.0/ This content is distributed under the terms of the Creative Commons Attribution 4.0 International license.

10.1128/msystems.00200-22.3 FIG S3 Shannon diversity index of urban and rural samples at the OTU level. The *P* value was from a Wilcoxon test. Download FIG S3, PDF file, 0.04 MB. Copyright © 2022 Sun et al. 2022 Sun et al. https://creativecommons.org/licenses/by/4.0/ This content is distributed under the terms of the Creative Commons Attribution 4.0 International license.

10.1128/msystems.00200-22.4 FIG S4 Taxonomic distribution of novel and known species-level OTUs associated with participant information. Each point is a species-level OTU. The *y* axis shows the Spearman’s correlation coefficients for age, physical activity, energy intake, fiber intake, and BMI, −log_10_(FDR) of ANOVA test for education, drinking water source, and toilet type and −log_10_(FDR ) × direction of change of Wilcoxon test for sex. The OTUs were colored based on their order classification, and insignificant OTUs are shown in light colors (FDR < 0.1). Download FIG S4, PDF file, 0.7 MB. Copyright © 2022 Sun et al. 2022 Sun et al. https://creativecommons.org/licenses/by/4.0/ This content is distributed under the terms of the Creative Commons Attribution 4.0 International license.

10.1128/msystems.00200-22.6 TABLE S1 Number of novel and known species-level OTUs significantly associated with participant information (FDR < 0.1). Significance was analyzed with Spearman’s correlation for age, physical activity, energy intake, fiber intake and BMI, ANOVA test for education, drinking water source and toilet type, and Wilcoxon test for sex. *P* values were adjusted with the Benjamini-Hochberg method for multiple tests. Download Table S1, PDF file, 0.04 MB. Copyright © 2022 Sun et al. 2022 Sun et al. https://creativecommons.org/licenses/by/4.0/ This content is distributed under the terms of the Creative Commons Attribution 4.0 International license.

### Functional differentiation between urban and rural microbiota.

**FIG 5:**
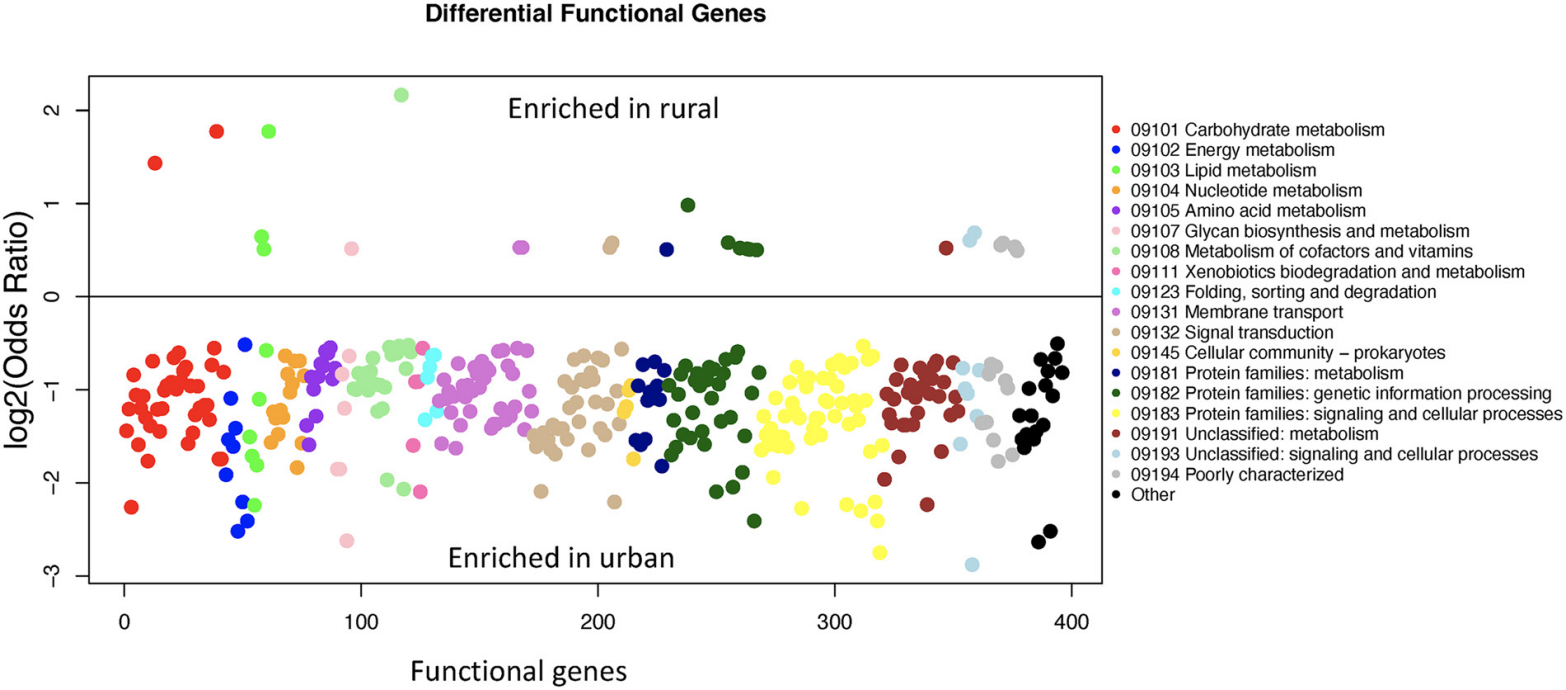
Differential functional genes between metagenome assembled genomes (MAGs) from rural and urban participants. The association of functional genes and urbanization was analyzed with Fisher’s exact test. Each point is a differential functional gene, and the *y* axis shows the log_2_ of odds ratio as shown in the figure. The *P* values were adjusted with the Benjamini-Hochberg method. Significance was determined with an FDR cutoff of 0.1. The genes were colored by their KEGG pathways.

**FIG 6:**
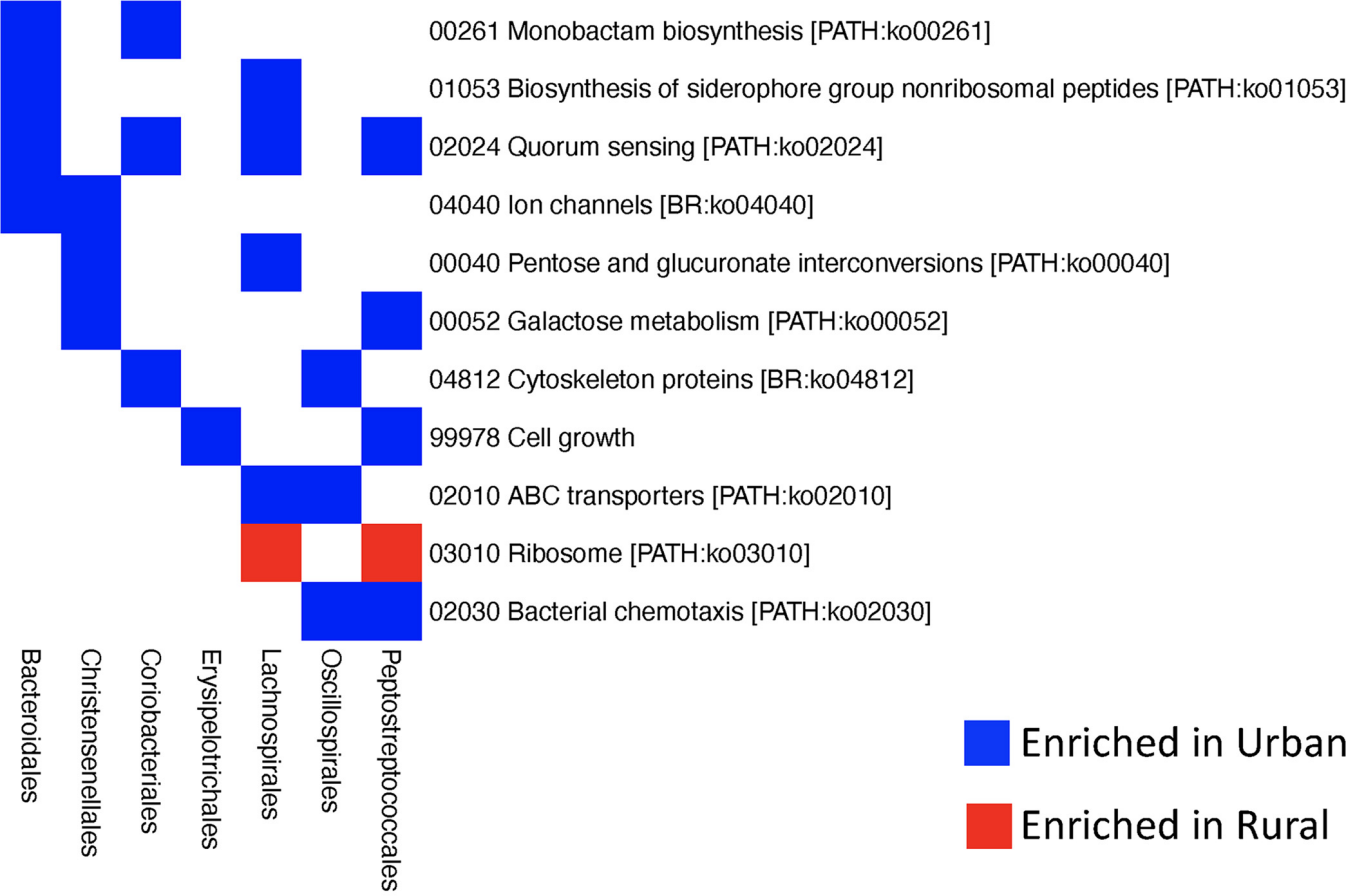
Consistent urbanization-associated pathway changes in multiple orders. The association between pathways and urbanization within each order was analyzed with Fisher’s exact test, and the significance was determined with an FDR cutoff of 0.1.

We annotated the functional genes of 843 high-quality MAGs with eggNOG-mapper, and on average, 1,280 genes were identified for each MAG, ranging from 866 to 3,509 genes. The MAGs from urban samples tend to have more genes than those assembled from rural samples (Fig. 1G). We tested whether the prevalence of each gene is associated with the urbanization status of samples, and this revealed 397 genes associated with urbanization (Fisher’s exact test, false-discovery rate [FDR] < 0.1), with 26 enriched in rural and 371 enriched in urban MAGs (Fig. 5). With the KEGG database, 85 of the differentially abundant genes were classified as transporters (02000 Transporters and 02010 ABC transporters), and 83 of them were enriched in urban samples, and 2 were enriched in rural samples. Genes involved in energy metabolism, nucleotide metabolism, amino acid metabolism, and xenobiotics biodegradation and metabolism were all enriched in urban samples. We also compared the functional genes of urban and rural MAGs that belong to the same order and observed some consistent changes across multiple orders (Fig. 6). Monobactams (monocyclic and bacterially produced β-lactam antibiotics) biosynthesis pathways were enriched, respectively, in urban genomes belonging to *Bacteroidales* and *Coriobacteriales*. Quorum sensing was enriched in urban genomes belonging to *Bacteroidales*, *Coriobacteriales*, *Lachnospirales*, and *Peptostreptococcales*. Biosynthesis of siderophore group nonribosomal peptides, ion channels, ABC transporters, and bacterial chemotaxis were all enriched in urban genomes of at least two orders, while the ribosome pathway was enriched in rural genomes.

We aligned the 843 high-quality genomes to the comprehensive antibiotic resistance protein homolog database (CARD) to estimate the presence of antibiotic resistance genes (ARGs) and found 404 ARGs (84 different ARGs) in 118 MAGs belonging to 44 species. The presence of ARGs was significantly higher in microbial genomes of urban origin (Fisher’s exact test, OR = 2.05, *P* = 0.00045), indicating that urbanization is associated with enrichment of individual microbial genomes consisting of ARGs. The most common ARGs are *rpoB*, *kdpE*, *acrB*, *acr*, *cpxA*, and *msbA*, which were also more common in urban microbiota. The MAGs classified as Escherichia flexneri, E. coli, and Klebsiella pneumoniae all consisted of ARGs, and the average numbers of ARGs are 20.9, 18.0, and 4.4, respectively. ARGs were found in the genomes of urban enriched pathogen Eggerthella lenta as well. ARGs were also very common in *Bifidobacterium* species, including Bifidobacterium longum (8 of 24 high-quality MAGs, 6 of urban origin and 2 of rural origin) and Bifidobacterium pseudocatenulatum (32 of 32 high-quality MAGs, 25 of urban origin and 7 of rural origin). Although the antibiotic resistance phenotypes have been reported for *Bifidobacteria* that are considered probiotics (17), the higher presence of ARGs in urban samples may raise a concern about the potentially increased ARG reservoir associated with urbanization. The increased ARGs in urban genomes suggest a hypothesis that the observed difference in genome size was mostly due to the higher number of ARGs. To evaluate this, we repeated the analysis removing the ARGs from the genomes and found that the urban genomes were still significantly larger than the rural genomes (Wilcoxon test, *P* = 2.4E-4) (Fig. S5), indicating that besides the ARGs, there are other factors contributing to the genome size.

10.1128/msystems.00200-22.5 FIG S5 Comparison of rural and urban genomes without ARGs. The *P* value was from a Wilcoxon test. Download FIG S5, PDF file, 0.2 MB. Copyright © 2022 Sun et al. 2022 Sun et al. https://creativecommons.org/licenses/by/4.0/ This content is distributed under the terms of the Creative Commons Attribution 4.0 International license.

## DISCUSSION

In this study, we used metagenomic assembly and binning approaches to reconstruct microbial genomes from gut metagenome samples of urban and rural communities in China. We recovered 2,518 high- and medium-quality genomes from those samples, among which 441 genomes (17.5%) could not be classified to the species level using GTDB-Tk with a combined method of tree placement, relative evolutionary divergence (RED), and ANI to reference genomes. The species-level OTUs clustered from these novel genomes increased the mapped reads from 47.6% to 65.3% across the samples. This indicates that a substantial proportion of microbial diversity has not yet been captured by reference databases in the gut microbiota of this population. This observation is consistent with previous studies that found less urbanized populations were enriched with novel microbial taxa (7, 9). The newly constructed genomes in this study contribute to ongoing efforts to expand the collection of microbial genomes of nonurbanized populations. Successful metagenomic assembly requires a high sequencing depth, and therefore, the genomes of rare taxa in studies such as ours are less likely to be successfully recovered. This may explain the remaining 35% unmapped reads in our study. Future studies with higher sequencing depth and further progress in algorithm development may allow for the future recovery of the genomes of rare taxa. While the extracted DNA of all samples was combined in equal aliquots for a single sequencing run, the resulting average sequencing reads per sample were 3.8% lower in rural samples than in urban samples. However, the assembled MAGs per sample were not significantly different between urban and rural samples. Therefore, we believe the small difference in sequencing depth is unlikely to have led to an unbalanced comparison of rural and urban samples. In addition, most of our analyses were performed at the individual MAG level, which presumably would not be sensitive to such differences.

The detailed view of the microbial genome that our study represents yielded both expected and unexpected observations about the differences between rural and urban microbial genomes. Consistent with published literature, we found that novel taxa were less prevalent and less abundant in urban populations than rural populations. The loss of novel species in the urban microbiota likely reflects the higher representation of the urbanized gut microbiota in current databases, while the rural communities harbored more novel diversity. Our study recruited participants from 41 communities in two provinces (Hunan and Guizhou) that vary by urbanization status. Because rural and urban samples were collected from multiple locations across the two provinces (including many in close geographic proximity), we think the observed rural and urban differences are unlikely an artifact of the difference between geographic locations. Our study further confirmed that urbanization is actively transforming the gut microbiota of local communities at the expense of loss and decline of microbes associated with traditional lifestyles. The loss and decline of the rural microbes in the urbanized population could be caused by a range of lifestyle changes as a result of urbanization, potentially diet changes that encouraged the growth of a different group of gut microbes. It is also possible that taxa that are acquired in rural (but not urban)-dwelling individuals are less represented in the current microbial genome databases. It is likely that the changes in the microbiome in urbanized populations results from a complex combination of multiple factors. We analyzed the host factors related to urbanization and microbial composition and found that education, drinking water source, toilet type, and energy and fiber intake were also associated with the similarity of the assembled genomes to the RefSeq database, indicating that the change in those factors may contribute to the urbanization-associated microbiome changes. However, these variables are associated with smaller changes than that observed for urbanization, suggesting that urbanization is a primary factor in driving our results.

We discovered the higher prevalence of taxa belonging to genera TM7x, Escherichia, and Streptococcus and species Streptococcus pasteurianus, Streptococcus parasanguinis, Rothia mucilaginosa, and Eggerthella lenta in our urban participants, which is of concern, as these taxa are well-known to include a number of pathogens or relate to infections (18, 19). Despite an inclusion criterion specifying no antibiotic use in the last 4 weeks before sampling, we found that the urban microbiota was enriched with genomes consisting of ARGs compared to rural microbiota. Unexpectedly, we also observed that the genomes in urban participants had a larger genome size than in rural participants, with this difference being small but statistically significant. Our results suggest that urbanization may be associated with an increase in pathogens and ARGs in the gut microbiota of local participants, indicating a higher risk for disease. It is possible that this increase in ARG density contributed partly to an overall increase in genome size. However, after removing identified ARGs, the urban genomes were still larger than rural genomes, indicating that factors other than ARGs may also have contributed to this change.

Our study revealed that urban genomes were generally of larger sizes and hence had a higher number of predicted ORFs. We found that a larger number of genes were enriched for the urban genomes, including 59 transporters and genes involved in energy metabolism and amino acid metabolism. However, it is difficult to determine whether these genes were truly enriched or the rural genomes were underannotated because of potential database bias. To control for this bias, we compared the genes of urban and rural genomes belonging to the same order and found consistent urban enrichment of monobactams biosynthesis pathway, biosynthesis of siderophore group nonribosomal peptides, quorum sensing, and ABC transporters across multiple orders, suggesting that these may not be caused by the database bias. However, this test was limited to order level and the more abundant orders due to lack of statistical power for a small number of genomes. The development of gene prediction approaches and better annotations of the newly constructed genomes will contribute to a better comparison between the rural and urban genomes.

Urbanization is associated with a large increase in the risk of inflammatory diseases. Our study leads to the natural hypothesis that the novel species we described in rural participants may play an important role in maintaining a healthy state of the gut microbiota. Under this model, loss of these protective taxa with urbanization leads to an increased presence of pathogens and gene transfer of ARGs, increasing the risk of disease. However, our study was not designed to analyze the separate influences of different aspects of changes in the urbanization process, such as changes in diet, physical activities, and environment. Future investigation controlling for specific changes during urbanization and modeling of the microbial dynamics associated with these aspects is important for a better understanding of these complex interactions.

## MATERIALS AND METHODS

### Metagenome sample collection and sequencing.

The metagenome samples in this study were collected as part of the China Health and Nutrition Survey (CHNS), which was designed to represent urban and rural communities in China and to understand the impact of urbanization on population health. The participants were recruited from 41 communities in 12 different prefecture-level divisions/counties in two provinces (Hunan and Guizhou) that vary by urbanization status. Among the 12 prefectures/counties, 8 sampled both urban and rural communities, with a geographic distance between these rural and urban communities within the same prefecture/county ranging from 2.4 to 168 km. Among the other four prefectures/counties, two were fully urban communities and two fully rural communities; the geographic distances between these urban and rural communities ranged from 2.4 km to 1,017 km. Both rural and urban samples were collected from multiple locations across the two provinces to reduce the influence of geographic locations on characterization of urbanization-related changes. A validated, multicomponent urbanization index (20) derived from household and community surveys (e.g., sanitation, transportation, housing infrastructure, social services) was used to define urbanization level. The study met the standards for the ethical treatment of participants and was approved by the Institutional Review Boards of the University of North Carolina at Chapel Hill and the National Institute of Health and Nutrition, Chinese Center for Disease Control and Prevention. Study participants gave informed consent. Study participants received adequate training and instructions for the collection process before collection. One stool sample was collected per participant. All collected samples were immediately frozen at −20°C, cold-chain transported to the central laboratory within 24 to 48 h, and frozen at −20°C until being processed. Participants with bowel disorder, diarrhea, recent antibiotics, or specific bacteria product intake in the last 4 weeks were not included in this study. We processed 214 samples for shotgun metagenome sequencing, including 113 from rural and 101 from urban participants. Stool DNA was extracted with Tiangen DNA extraction kits (Tiangen Biotech, Beijing, China) following the instructions. The extracted DNA was processed and sequenced as 150PE reads on an Illumina platform at Novogene Bioinformatics Technology (Tianjin, China).

### Genome assembly and quality control.

Human contamination in the shotgun metagenome sequences was removed with Kneaddata, and low-quality reads were removed with the default parameters of Trimmomatic (read length, <50% of total input read length; cut reads once the average quality score of 4 bases was <20) (21). The genome assembly and binning pipeline follow the methods previously published (10). After filtering, all samples were individually assembled with metaSPAdes (v3.13.0) (22). The resulting contigs >2,000 bp were binned with MetaBAT 2 (v2.12.1) using default parameters (23). The quality of assembled genomes was estimated with CheckM with the lineage_wf workflow, including completeness, contamination, and strain heterogeneity. The quality of MAGs was determined based on the metagenome-assembled genome (MIMAG) criteria (high-quality, completeness > 90%, contamination < 5%, strain heterogeneity 0; medium quality, completeness > 50%, contamination < 5%) (24). MAGs with completeness <50% or contamination >5% were excluded from the following analyses. Mash (12) was used to calculate the distances between the identified MAGs and the NCBI RefSeq genome database (release 93, March 2019).

### Taxonomic annotation, pangenome abundance, functional characterization, and statistical analysis.

MAGs that passed quality control were annotated with the taxonomy classification tool GTDB-Tk (v0.3.2) (25) with the workflow classify_wf based on the Genome Taxonomy Database (release 89) (26). We then constructed pangenomes for the MAGs considered the same species. Known MAGs classified to the same species by GTDB-Tk were grouped, and their genes were clustered at a 90% DNA identity to pangenomes with vsearch (v2.14.1) (27). MAGs that were unclassified at the species level were grouped at the lowest taxonomic rank, and FastANI (28) was used to calculate the average nucleotide identity (ANI) between each pair of MAGs. MAGs with ANIs of >95% were grouped, and their genes were clustered to pangenomes as well with a 90% similarity threshold. The prevalence and abundance of pangenomes were estimated by mapping them to the shotgun metagenome sequencing reads with BWA-MEM and CoverM (v0.3.1) (29).

Only functional genes of high-quality MAGs were annotated to reduce the impact of incomplete genomes on gene contents. Open reading frames of the MAGs were predicted with OrfM (v0.7.1) (30) and then annotated with EggNOG mapper (v1.0.3) (31) based on the EggNOG database (32). The KEGG annotations and the associated Brite hierarchy system were primarily used in this study. The presence of antibiotic resistance genes (ARG) was estimated by aligning the high-quality genomes to the comprehensive antibiotic resistance protein homolog database (CARD) using BLAST with a cutoff of 90% identity and 100-bp alignment (33, 34).

Statistical significance was determined with Wilcoxon test and Fisher’s exact tests. *P* values were adjusted for multiple hypotheses testing with Benjamini-Hochberg method. Significance was determined as FDR of <0.1 when adjusted for multiple-hypotheses testing and a *P* value of <0.05 when a single test was performed. All statistical tests were performed with R (v3.5.1). Missing data were not included in the analysis. Scripts used in this study and a STROMs checklist were available at https://github.com/ssun6/MAG_urbanization.git.

### Data availability.

Data used in this study are available upon request to Shufa Du (chns@unc.edu). We cannot deposit the data to a public database due to governmental regulations and restrictions on the release of human bio-omics data in China.

## References

[1] United Nations Department of Economic and Social Affairs. 2018. World urbanization prospects: the 2018 revision. United Nations Department of Economic and Social Affairs, New York, NY.

[2] Carrillo-Larco RM, Bernabé-Ortiz A, Pillay TD, Gilman RH, Sanchez JF, Poterico JA, Quispe R, Smeeth L, Miranda JJ. 2016. Obesity risk in rural, urban and rural-to-urban migrants: prospective results of the PERU MIGRANT study. Int J Obes (Lond) 40:181–185. doi:10.1038/ijo.2015.140. 26228458 PMC4677453

[3] Zhou M, Astell-Burt T, Yin P, Feng X, Page A, Liu Y, Liu J, Li Y, Liu S, Wang L, Wang L, Wang L. 2015. Spatiotemporal variation in diabetes mortality in China: multilevel evidence from 2006 and 2012. BMC Public Health 15:633. doi:10.1186/s12889-015-1982-0. 26159911 PMC4496807

[4] Song H-N, Go S-I, Lee WS, Kim Y, Choi HJ, Lee US, Kang MH, Lee G-W, Kim H-G, Kang JH, Kang YS, Lee J-H, Jung J-M, Hong SC. 2016. Population-based regional cancer incidence in Korea: comparison between urban and rural areas. Cancer Res Treat 48:789–797. doi:10.4143/crt.2015.062. 26194369 PMC4843717

[5] Mathenge W, Foster A, Kuper H. 2010. Urbanization, ethnicity and cardiovascular risk in a population in transition in Nakuru, Kenya: a population-based survey. BMC Public Health 10:569. doi:10.1186/1471-2458-10-569. 20860807 PMC2956724

[6] Yatsunenko T, Rey FE, Manary MJ, Trehan I, Dominguez-Bello MG, Contreras M, Magris M, Hidalgo G, Baldassano RN, Anokhin AP, Heath AC, Warner B, Reeder J, Kuczynski J, Caporaso JG, Lozupone CA, Lauber C, Clemente JC, Knights D, Knight R, Gordon JI. 2012. Human gut microbiome viewed across age and geography. Nature 486:222–227. doi:10.1038/nature11053. 22699611 PMC3376388

[7] Vangay P, Johnson AJ, Ward TL, Al-Ghalith GA, Shields-Cutler RR, Hillmann BM, Lucas SK, Beura LK, Thompson EA, Till LM, Batres R, Paw B, Pergament SL, Saenyakul P, Xiong M, Kim AD, Kim G, Masopust D, Martens EC, Angkurawaranon C, McGready R, Kashyap PC, Culhane-Pera KA, Knights D. 2018. US immigration westernizes the human gut microbiome. Cell 175:962–972.e910. doi:10.1016/j.cell.2018.10.029. 30388453 PMC6498444

[8] Winglee K, Howard AG, Sha W, Gharaibeh RZ, Liu J, Jin D, Fodor AA, Gordon-Larsen P. 2017. Recent urbanization in China is correlated with a Westernized microbiome encoding increased virulence and antibiotic resistance genes. Microbiome 5:121. doi:10.1186/s40168-017-0338-7. 28915922 PMC5603068

[9] Pasolli E, Asnicar F, Manara S, Zolfo M, Karcher N, Armanini F, Beghini F, Manghi P, Tett A, Ghensi P, Collado MC, Rice BL, DuLong C, Morgan XC, Golden CD, Quince C, Huttenhower C, Segata N. 2019. Extensive unexplored human microbiome diversity revealed by over 150,000 genomes from metagenomes spanning age, geography, and lifestyle. Cell 176:649–662.e620. doi:10.1016/j.cell.2019.01.001. 30661755 PMC6349461

[10] Almeida A, Mitchell AL, Boland M, Forster SC, Gloor GB, Tarkowska A, Lawley TD, Finn RD. 2019. A new genomic blueprint of the human gut microbiota. Nature 568:499–504. doi:10.1038/s41586-019-0965-1. 30745586 PMC6784870

[11] Nayfach S, Shi ZJ, Seshadri R, Pollard KS, Kyrpides NC. 2019. New insights from uncultivated genomes of the global human gut microbiome. Nature 568:505–510. doi:10.1038/s41586-019-1058-x. 30867587 PMC6784871

[12] Ondov BD, Treangen TJ, Melsted P, Mallonee AB, Bergman NH, Koren S, Phillippy AM. 2016. Mash: fast genome and metagenome distance estimation using MinHash. Genome Biol 17:132. doi:10.1186/s13059-016-0997-x. 27323842 PMC4915045

[13] Li Y, Chen X, Zhang Z, Wang L, Wang J, Zeng J, Yang J, Lu B. 2019. Microbiological and clinical characteristics of Streptococcus gallolyticus subsp. pasteurianus infection in China. BMC Infect Dis 19:791. doi:10.1186/s12879-019-4413-5. 31500570 PMC6734276

[14] Geng J, Chiu C-H, Tang P, Chen Y, Shieh H-R, Hu S, Chen Y-YM. 2012. Complete genome and transcriptomes of Streptococcus parasanguinis FW213: phylogenic relations and potential virulence mechanisms. PLoS One 7:e34769. doi:10.1371/journal.pone.0034769. 22529932 PMC3329508

[15] Ramanan P, Barreto JN, Osmon DR, Tosh PK. 2014. Rothia bacteremia: a 10-year experience at Mayo Clinic, Rochester, Minnesota. J Clin Microbiol 52:3184–3189. doi:10.1128/JCM.01270-14. 24951810 PMC4313135

[16] Gardiner BJ, Tai A, Kotsanas D, Francis MJ, Roberts SA, Ballard SA, Junckerstorff RK, Korman TM. 2015. Clinical and microbiological characteristics of Eggerthella lenta bacteremia. J Clin Microbiol 53:626–635. doi:10.1128/JCM.02926-14. 25520446 PMC4298500

[17] Gueimonde M, Sánchez B, de los Reyes-Gavilán CG, Margolles A. 2013. Antibiotic resistance in probiotic bacteria. Front Microbiol 4:202. doi:10.3389/fmicb.2013.00202. 23882264 PMC3714544

[18] He X, McLean JS, Edlund A, Yooseph S, Hall AP, Liu S-Y, Dorrestein PC, Esquenazi E, Hunter RC, Cheng G, Nelson KE, Lux R, Shi W. 2015. Cultivation of a human-associated TM7 phylotype reveals a reduced genome and epibiotic parasitic lifestyle. Proc Natl Acad Sci USA 112:244–249. doi:10.1073/pnas.1419038112. 25535390 PMC4291631

[19] Krzyściak W, Pluskwa K, Jurczak A, Kościelniak D. 2013. The pathogenicity of the Streptococcus genus. Eur J Clin Microbiol Infect Dis 32:1361–1376. doi:10.1007/s10096-013-1914-9. 24141975 PMC3824240

[20] Jones-Smith JC, Popkin BM. 2010. Understanding community context and adult health changes in China: development of an urbanicity scale. Soc Sci Med 71:1436–1446. doi:10.1016/j.socscimed.2010.07.027. 20810197 PMC2942954

[21] Bolger AM, Lohse M, Usadel B. 2014. Trimmomatic: a flexible trimmer for Illumina sequence data. Bioinformatics 30:2114–2120. doi:10.1093/bioinformatics/btu170. 24695404 PMC4103590

[22] Nurk S, Meleshko D, Korobeynikov A, Pevzner PA. 2017. metaSPAdes: a new versatile metagenomic assembler. Genome Res 27:824–834. doi:10.1101/gr.213959.116. 28298430 PMC5411777

[23] Kang DD, Li F, Kirton E, Thomas A, Egan R, An H, Wang Z. 2019. MetaBAT 2: an adaptive binning algorithm for robust and efficient genome reconstruction from metagenome assemblies. PeerJ. 7:e7359. doi:10.7717/peerj.7359. 31388474 PMC6662567

[24] Bowers RM, Kyrpides NC, Stepanauskas R, Harmon-Smith M, Doud D, Reddy TBK, Schulz F, Jarett J, Rivers AR, Eloe-Fadrosh EA, Tringe SG, Ivanova NN, Copeland A, Clum A, Becraft ED, Malmstrom RR, Birren B, Podar M, Bork P, Weinstock GM, Garrity GM, Dodsworth JA, Yooseph S, Sutton G, Glöckner FO, Gilbert JA, Nelson WC, Hallam SJ, Jungbluth SP, Ettema TJG, Tighe S, Konstantinidis KT, Liu W-T, Baker BJ, Rattei T, Eisen JA, Hedlund B, McMahon KD, Fierer N, Knight R, Finn R, Cochrane G, Karsch-Mizrachi I, Tyson GW, Rinke C, Lapidus A, Meyer F, Yilmaz P, Parks DH, Eren AM, et al. 2017. Minimum information about a single amplified genome (MISAG) and a metagenome-assembled genome (MIMAG) of bacteria and archaea. Nat Biotechnol 35:725–731. doi:10.1038/nbt.3893. 28787424 PMC6436528

[25] Chaumeil P-A, Mussig AJ, Hugenholtz P, Parks DH. 2020. GTDB-Tk: a toolkit to classify genomes with the Genome Taxonomy Database. Bioinformatics 36:1925–1927. doi:10.1093/bioinformatics/btz848. PMC7703759 31730192

[26] Parks DH, Chuvochina M, Waite DW, Rinke C, Skarshewski A, Chaumeil P-A, Hugenholtz P. 2018. A standardized bacterial taxonomy based on genome phylogeny substantially revises the tree of life. Nat Biotechnol 36:996–1004. doi:10.1038/nbt.4229. 30148503

[27] Rognes T, Flouri T, Nichols B, Quince C, Mahé F. 2016. VSEARCH: a versatile open source tool for metagenomics. PeerJ 4:e2584. doi:10.7717/peerj.2584. 27781170 PMC5075697

[28] Jain C, Rodriguez-R LM, Phillippy AM, Konstantinidis KT, Aluru S. 2018. High throughput ANI analysis of 90K prokaryotic genomes reveals clear species boundaries. Nat Commun 9:5114. doi:10.1038/s41467-018-07641-9. 30504855 PMC6269478

[29] Li H. 2013. Aligning sequence reads, clone sequences and assembly contigs with BWA-MEM. arXiv 1303.3997v2 [q-bio.GN]. https://arxiv.org/abs/1303.3997.

[30] Woodcroft BJ, Boyd JA, Tyson GW. 2016. OrfM: a fast open reading frame predictor for metagenomic data. Bioinformatics 32:2702–2703. doi:10.1093/bioinformatics/btw241. 27153669 PMC5013905

[31] Huerta-Cepas J, Forslund K, Coelho LP, Szklarczyk D, Jensen LJ, Von Mering C, Bork P. 2017. Fast genome-wide functional annotation through orthology assignment by eggNOG-Mapper. Mol Biol Evol 34:2115–2122. doi:10.1093/molbev/msx148. 28460117 PMC5850834

[32] Huerta-Cepas J, Szklarczyk D, Heller D, Hernández-Plaza A, Forslund SK, Cook H, Mende DR, Letunic I, Rattei T, Jensen LJ, von Mering C, Bork P. 2019. eggNOG 5.0: a hierarchical, functionally and phylogenetically annotated orthology resource based on 5090 organisms and 2502 viruses. Nucleic Acids Res 47:D309–D314. doi:10.1093/nar/gky1085. 30418610 PMC6324079

[33] Li B, Yang Y, Ma L, Ju F, Guo F, Tiedje JM, Zhang T. 2015. Metagenomic and network analysis reveal wide distribution and co-occurrence of environmental antibiotic resistance genes. ISME J 9:2490–2502. doi:10.1038/ismej.2015.59. 25918831 PMC4611512

[34] Pal C, Bengtsson-Palme J, Kristiansson E, Larsson DJ. 2016. The structure and diversity of human, animal and environmental resistomes. Microbiome 4:54. doi:10.1186/s40168-016-0199-5. 27717408 PMC5055678

