# Supplementary material for: Loss of Novel Diversity in Human Gut Microbiota Associated with Ongoing Urbanization in China
Source: mSystems. 2022 Jun 21;7(4):e00200-22. doi: 10.1128/msystems.00200-22 (PMC9426419; doi:10.1128/msystems.00200-22)
Supplement: FIG S1 [file msystems.00200-22-s0001.pdf]

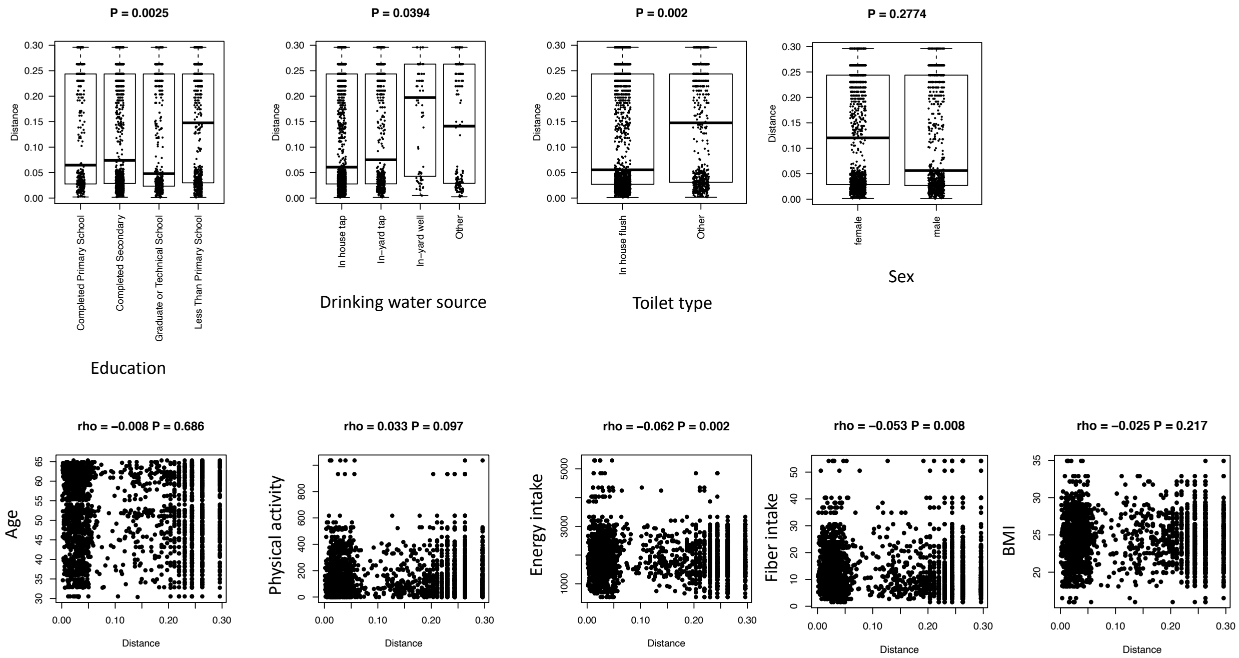

Fig. S1. The associations between urbanization related factors and the mash distances to database. The associations between mash distance (see methods) and education, drinking water source and toilet type were tested with ANOVA. The association between mash distance and sex was tested with Wilcoxon test. The associations between mash distance and age, physical activity, energy intake, fiber intake and BMI were tested with Spearman’s correlation.
