# Supplementary figures and images for: Loss of Novel Diversity in Human Gut Microbiota Associated with Ongoing Urbanization in China
Source: mSystems. 2022 Jun 21;7(4):e00200-22. doi: 10.1128/msystems.00200-22 (PMC9426419; doi:10.1128/msystems.00200-22)

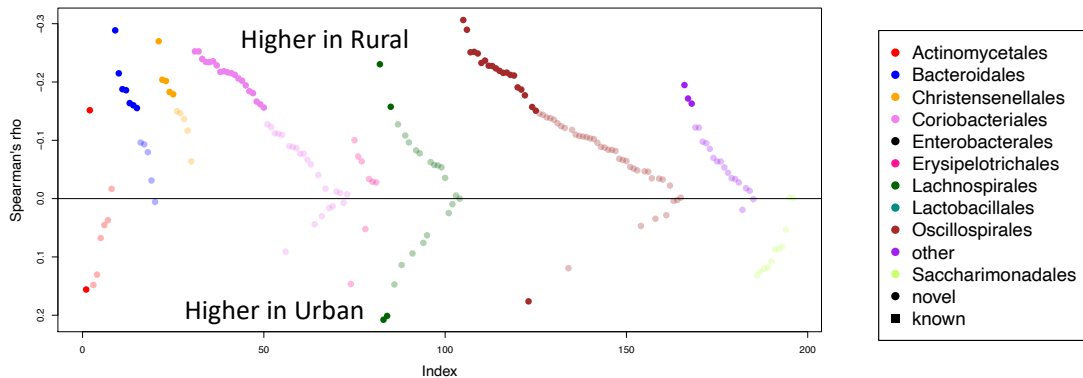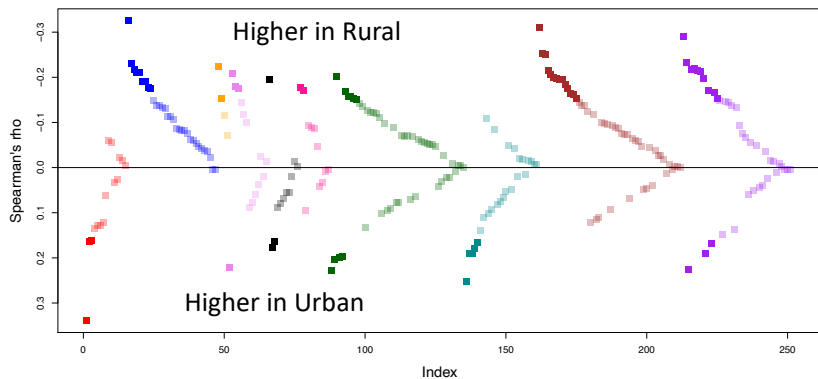

Supplement: FIG S2 [file msystems.00200-22-s0002.pdf]

**P = 0.023**

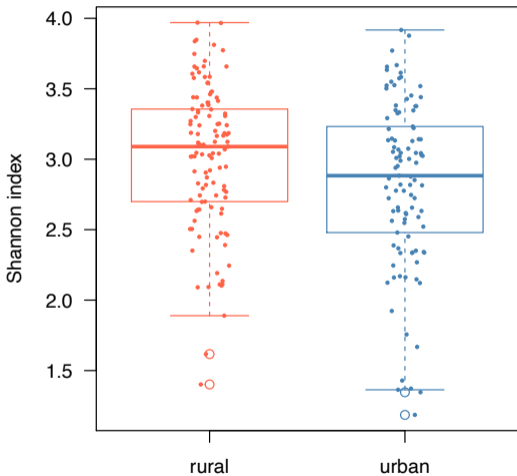

Supplement: FIG S3 [file msystems.00200-22-s0003.pdf]

**P = 0.00024**

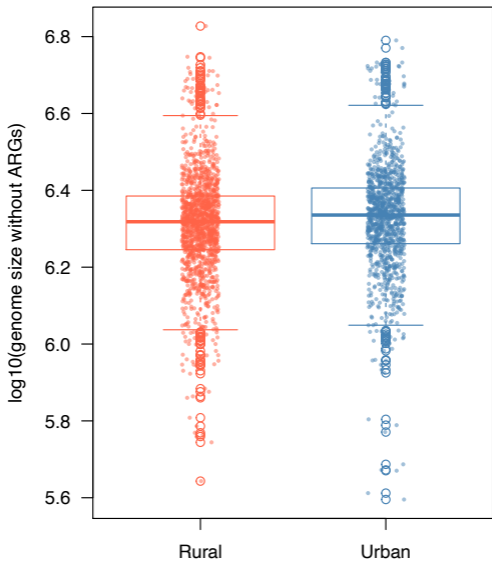

Supplement: FIG S5 [file msystems.00200-22-s0005.pdf]
