# Supplementary material for: Loss of Novel Diversity in Human Gut Microbiota Associated with Ongoing Urbanization in China
Source: mSystems. 2022 Jun 21;7(4):e00200-22. doi: 10.1128/msystems.00200-22 (PMC9426419; doi:10.1128/msystems.00200-22)
Supplement: FIG S4 [file msystems.00200-22-s0004.pdf]

Age

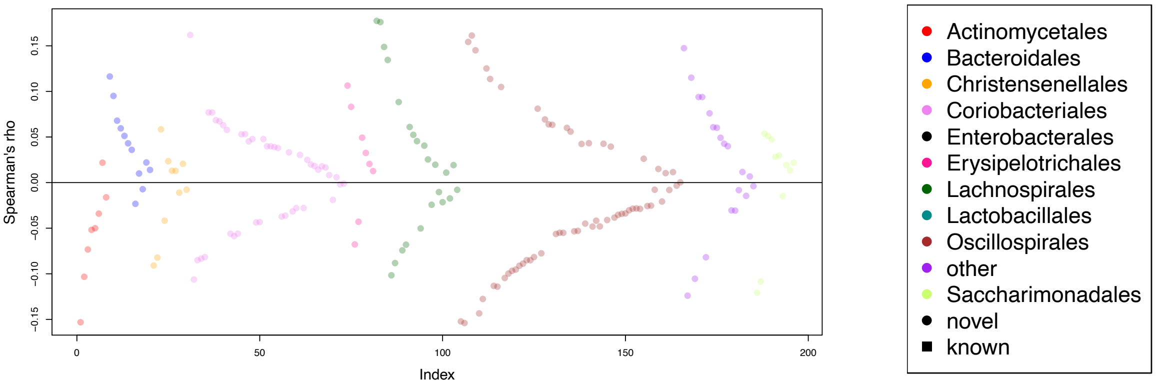

Spearman's rho
Index
Actinomycetales
Bacteroidales
Christensenellales
Coriobacteriales
Enterobacterales
Erysipelotrichales
Lachnospirales
Lactobacillales
Oscillospirales
other
Saccharimonadales
novel
known

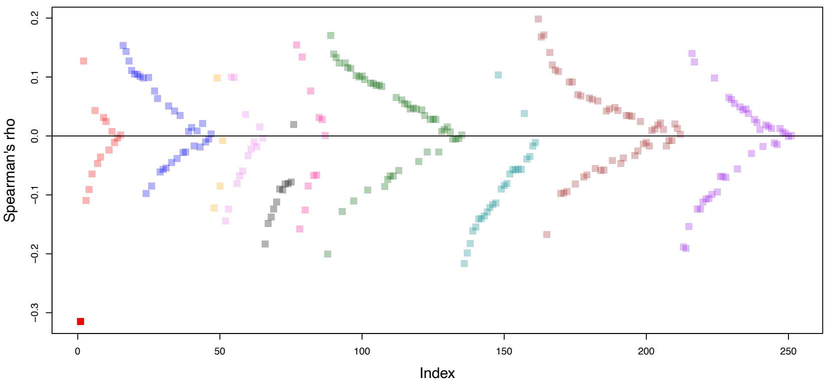

Spearman's rho
Index

Physical activity

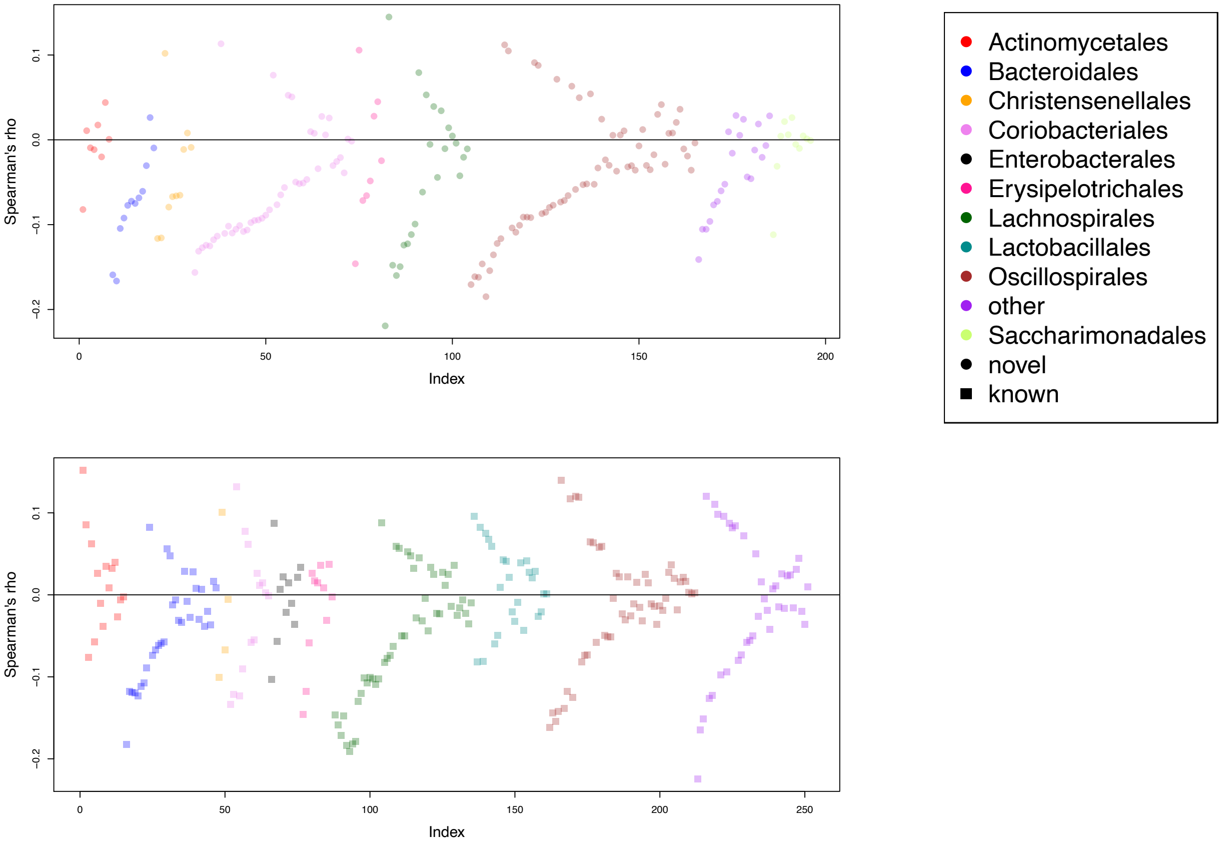

Energy intake

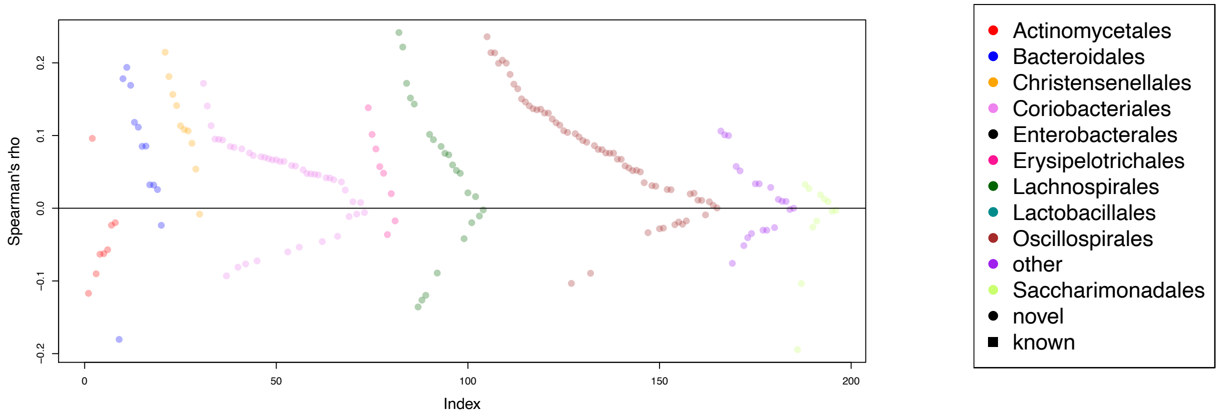

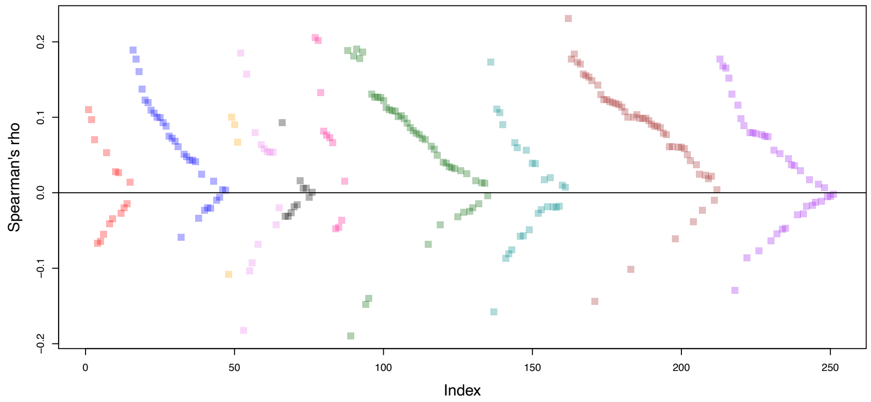

Fiber intake

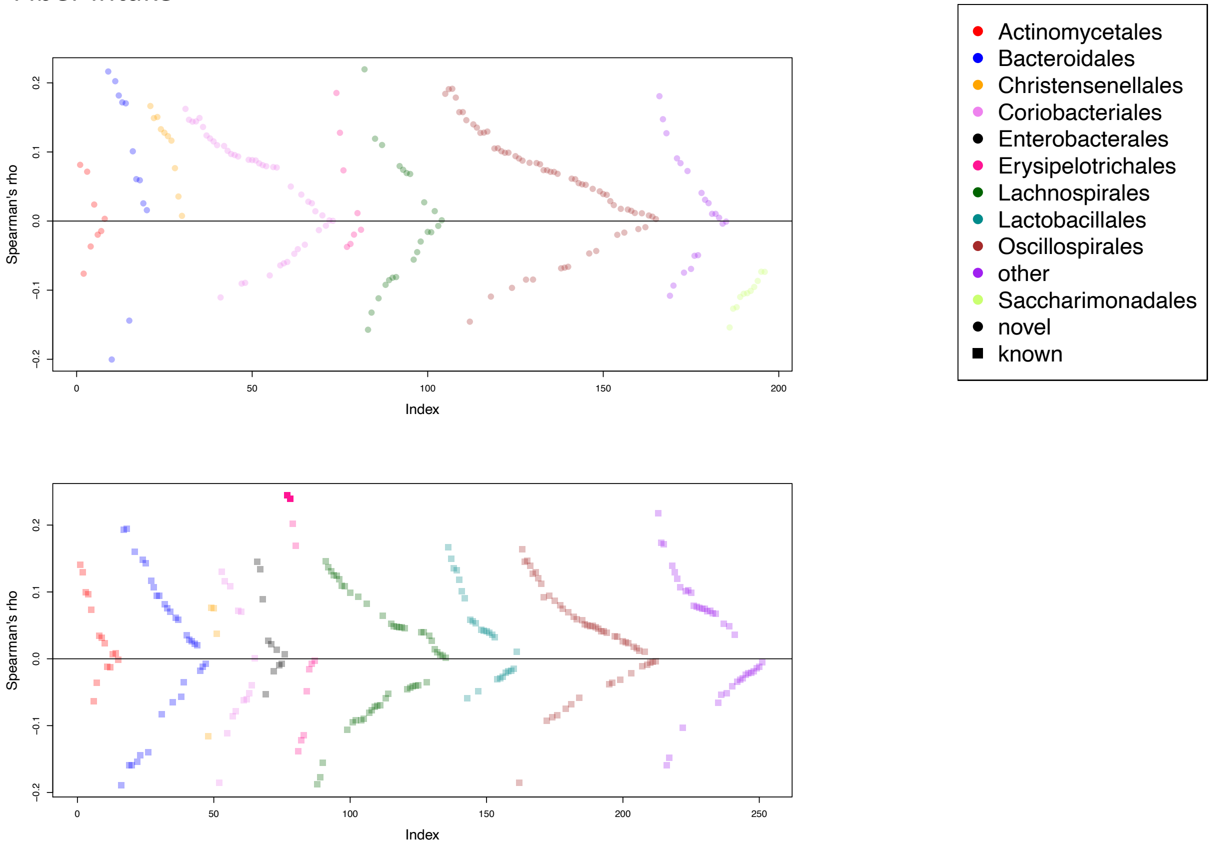

BMI

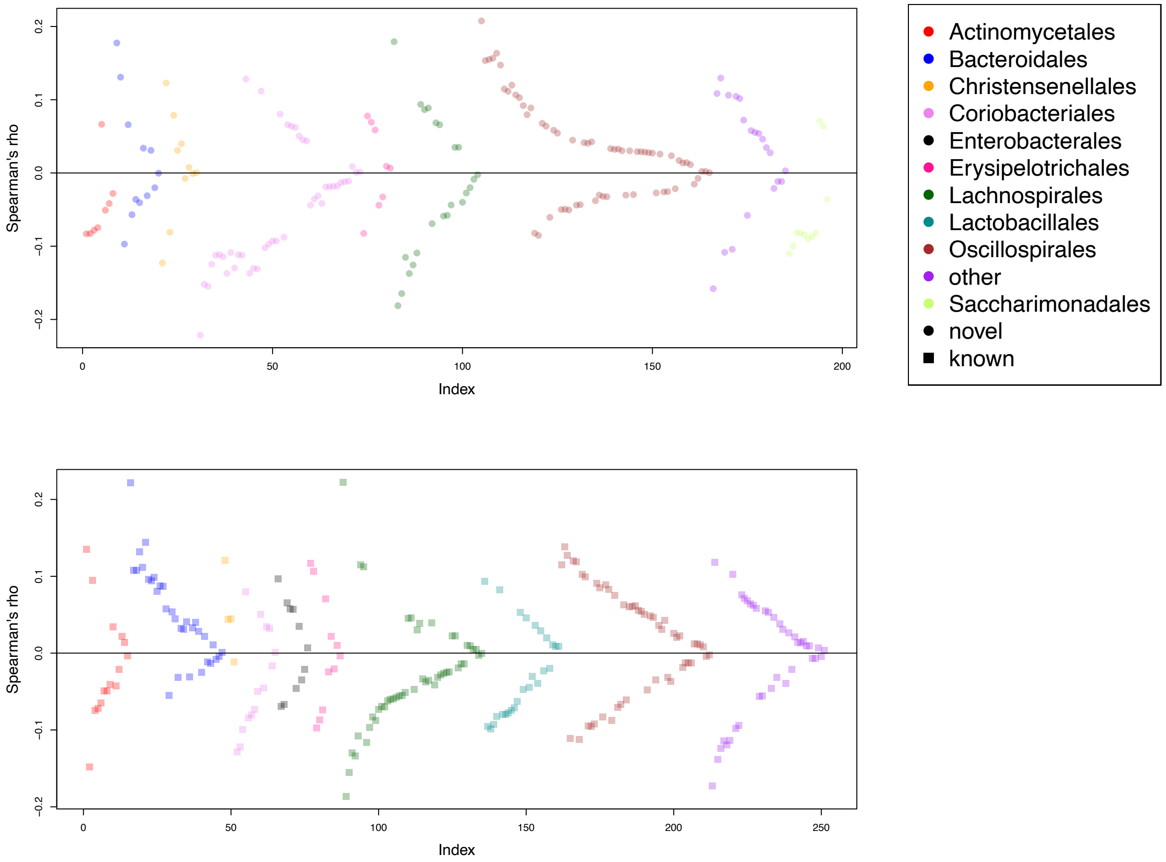

Spearman's rho
Index
Actinomycetales
Bacteroidales
Christensenellales
Coriobacteriales
Enterobacterales
Erysipelotrichales
Lachnospirales
Lactobacillales
Oscillospirales
other
Saccharimonadales
novel
known

Sex

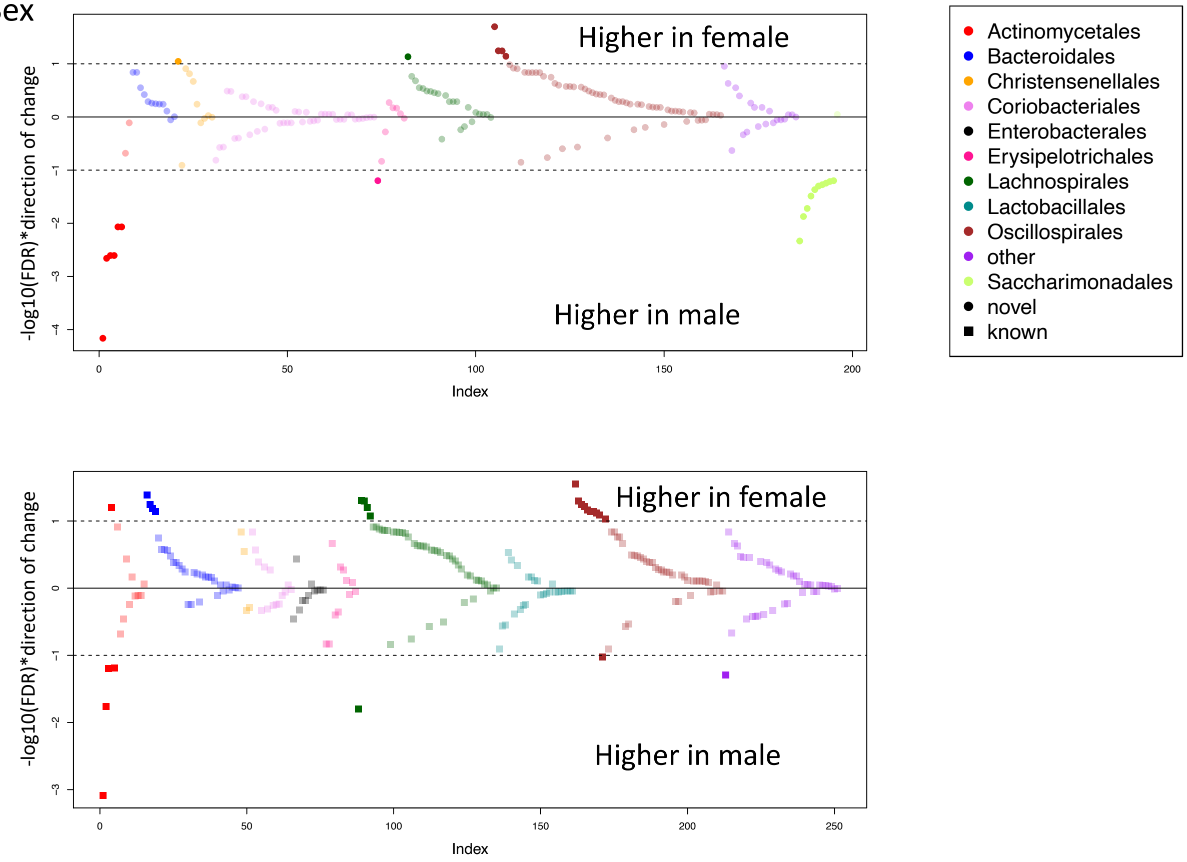

Higher in female
Higher in male
-log10(FDR)*direction of change
Index
Actinomycetales
Bacteroidales
Christensenellales
Coriobacteriales
Enterobacterales
Erysipelotrichales
Lachnospirales
Lactobacillales
Oscillospirales
other
Saccharimonadales
novel
known
Higher in female
Higher in male
-log10(FDR)*direction of change
Index

Education

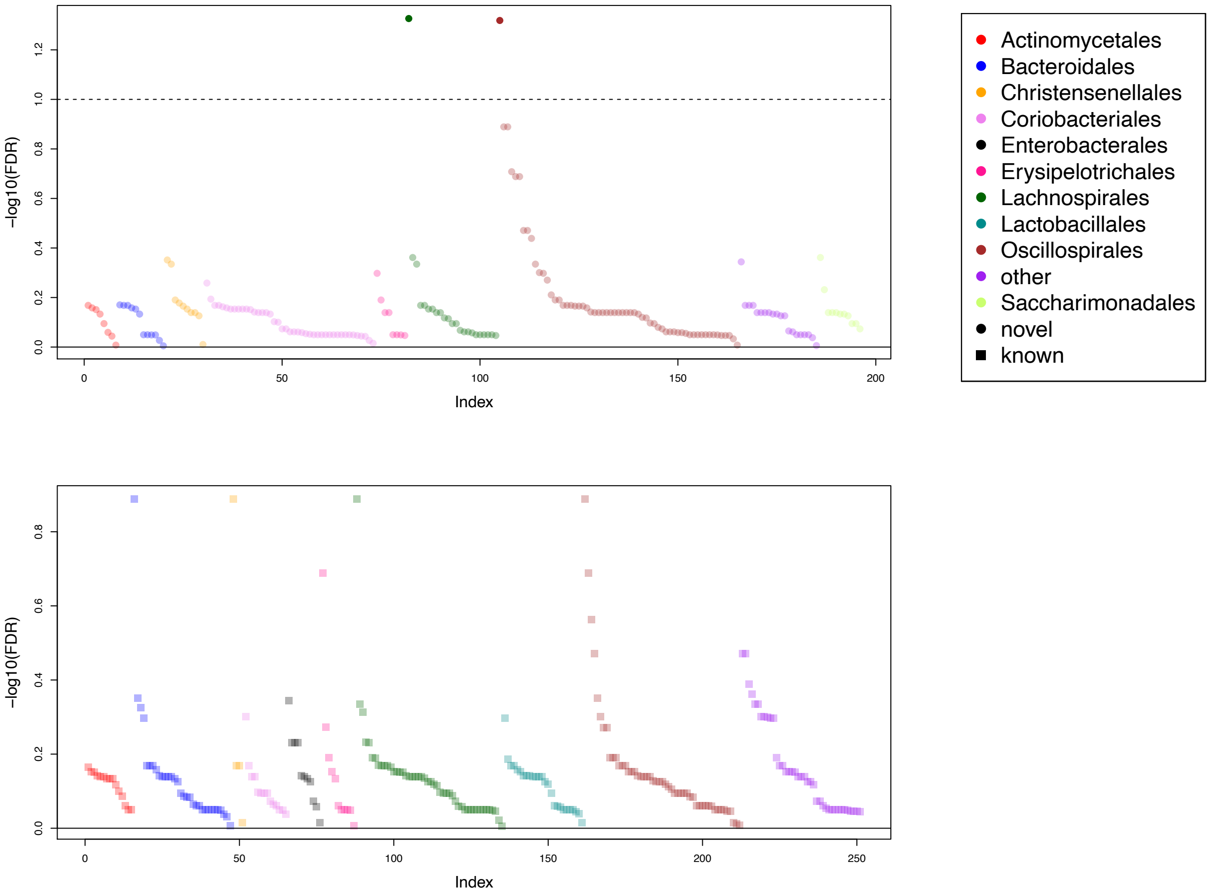

−log10(FDR)
Index
Actinomycetales
Bacteroidales
Christensenellales
Coriobacteriales
Enterobacterales
Erysipelotrichales
Lachnospirales
Lactobacillales
Oscillospirales
other
Saccharimonadales
novel
known

Drinking water source

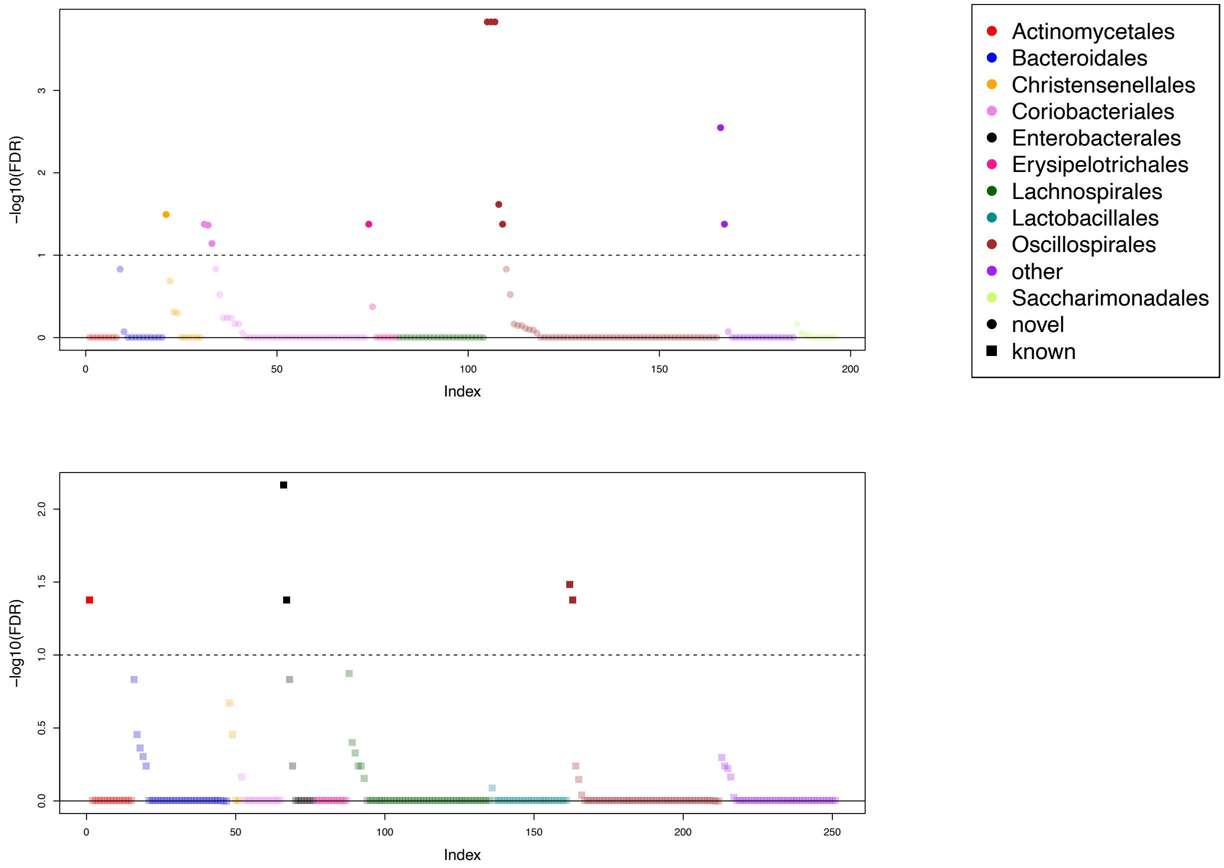

Toilet type

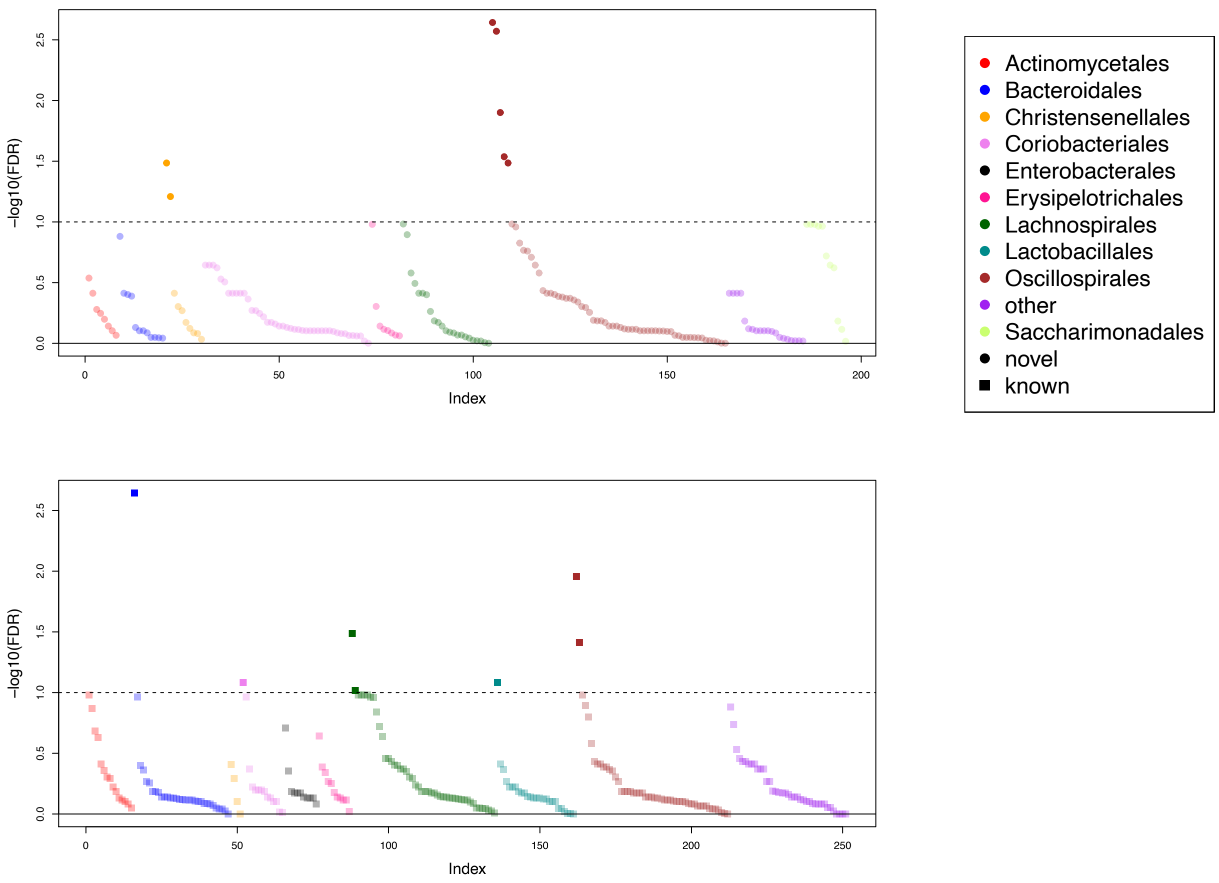

−log10(FDR)
Index
Actinomycetales
Bacteroidales
Christensenellales
Coriobacteriales
Enterobacterales
Erysipelotrichales
Lachnospirales
Lactobacillales
Oscillospirales
other
Saccharimonadales
novel
known
