# Supplementary material for: Loss of Novel Diversity in Human Gut Microbiota Associated with Ongoing Urbanization in China
Source: mSystems. 2022 Jun 21;7(4):e00200-22. doi: 10.1128/msystems.00200-22 (PMC9426419; doi:10.1128/msystems.00200-22)
Supplement: TABLE S1 [file msystems.00200-22-s0006.pdf]

Table S1. The number of novel and known species-level OTUs significantly associated with participant information (FDR<0.1). The significance was analyzed with Spearman's correlation for age, physical activity, energy intake, fiber intake and BMI, ANOVA test for education, drinking water source and toilet type, and Wilcoxon test for sex. P values were adjusted with the Benjamini-Hochberg method for multiple tests.

	Novel	Known
Age	0	1
Sex	23	26
Education	2	0
Physical activity	0	0
BMI	0	0
Fiber intake	0	2
Energy intake	0	0
Drinking water source	12	5
Toilet type	7	7
